# Experiences of facilitators and barriers for fulfilment of human needs when living with restless legs syndrome: a qualitative study

**DOI:** 10.1080/17482631.2024.2348884

**Published:** 2024-05-12

**Authors:** Elzana Odzakovic, Monika Allgurin, Lise-Lotte Jonasson, Sandra Öberg, Bengt Fridlund, Martin Ulander, Jonas Lind, Anders Broström

**Affiliations:** a School of Health and Welfare, Jönköping University, Jönköping, Sweden; b Centre for Interprofessional Collaboration within Emergency Care (CICE), Linnaeus University, Växjö, Sweden; c Department of Clinical Neurophysiology, Linköping University Hospital, Linköping, Sweden; d Department of Biomedical and Clinical Sciences, Division of Neurobiology, Linköping University, Linköping, Sweden; e Section of Neurology, Department of Internal Medicine, County Hospital Ryhov, Jönköping, Sweden; f Department of Health and Caring Sciences, Western Norway University of Applied Sciences, Bergen, Norway

**Keywords:** Human needs, patient-centred, qualitative content analysis, restless legs syndrome, Willis Ekbom disease, Wittmaack Ekbom syndrome

## Abstract

**Purpose:**

Restless Legs Syndrome (RLS) is a widespread condition that affects sleep leading to daytime sleepiness, depression, and reduced quality of life. This study aims to determine and describe how patients with RLS experience their everyday life, with a focus on facilitators and barriers related to Maslow’s hierarchical theory of human needs.

**Method:**

Semi-structured interviews were analysed with qualitative content analysis resulting in facilitators and barriers affecting the fulfilment of the five human needs.

**Results:**

Addressing RLS symptoms through medications and a quiet sleep environment fulfils psychological needs. Control over RLS symptoms, engagement in activities, trust in treatments, and social support meet safety and security needs. Social inclusion, close relationships, and meaningful interactions fulfil a sense of belongingness and love needs despite RLS. Competence in managing RLS, effective self-care strategies, confident communication, and trust-building support esteem needs. Finally, comprehensive understanding through person-centred interventions and coping fulfils the self-actualization needs in managing RLS.

**Conclusion:**

Holistic and person-centred interventions, including facilitators for the fulfilment of physiological, psychological, and social needs could help healthcare professionals to provide holistic care.

## Introduction

Restless legs syndrome (RLS) is a highly prevalent sensory-motor disorder, impacting approximately 3% of the global population (Broström et al., 2023), with a circadian rhythm profile, characterized by an urge to move the arms and legs, usually associated with discomfort, pain, and motor restlessness. The complete pathophysiology is not known, but genetic variants, abnormal iron metabolism, dopaminergic and central opioid system dysfunction are seen as potential mechanisms (Khachatryan et al., 2022). The diagnosis, based on five essential criteria (IRLSSG; Allen et al., 2014) (Table I), is commonly made in primary care, but the variation and fluctuation in symptoms, signs, and symptom burden, also when treatment is initiated, make RLS a difficult condition to diagnose and treat (Garcia-Borreguero et al., 2018). Consequently, this may result in underdiagnosis, but at the same time, there is also a risk for overdiagnosis because several symptoms are non-specific and can be related to other conditions. An individually adapted intermittent pharmacological treatment is commonly prescribed after lifestyle change, medication effect and iron supplementation have been considered (Lv et al., 2021). Several drugs are available (i.e., dopamine agonists, L-dopa, alpha-2-delta ligands, opioids, or iron), with dopaminergic agents considered to be the first-line treatment. Dopamine agonists are often used but can cause increased RLS symptoms (i.e., augmentation) affecting the everyday life of the patient (Winkelman, 2022). The additive effect of self-care interventions (Harrison et al., 2019) has been evaluated in only a few studies, which makes it difficult to assess their effects. To further advance the qualitative research in the field of RLS, and to build upon the accumulating evidence base, we need to understand patients´ experiences and how they manage their symptoms in relation to various human needs in everyday life.

**Table I.** Diagnostic criteria established by the international restless legs syndrome study group (2014).

Essential diagnostic criteria	
Criteria I	Desire to move arms and legs usually associated with discomfort.
Criteria II	Motor restlessness
Criteria III	Symptoms are worse or exclusively present at rest with at least partial and temporary relief by activity
Criteria IV	Symptoms are worse in the evening/night
Criteria V	The occurrence of these symptoms is not only reported as symptoms primary to another medical/behavioural condition, but may also be secondary to other diseases or conditions

**Figure 1.**
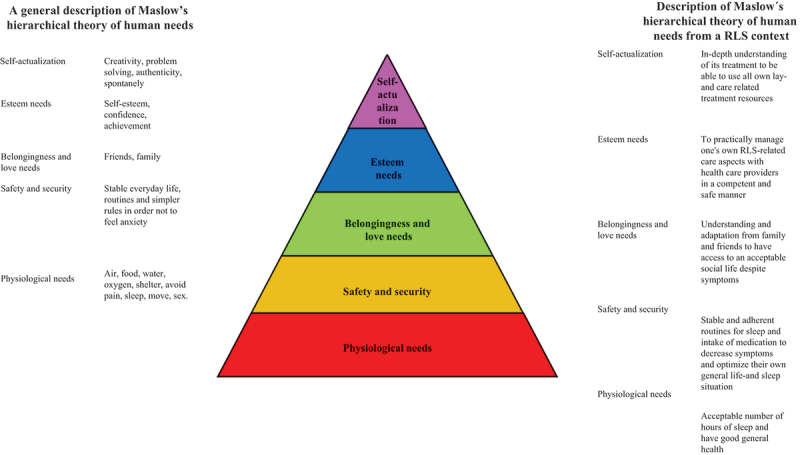
Maslow’s hierarchical pyramid of human needs.

When exploring the clinical presentation of RLS (Didato et al., 2020), existing evidence has mostly focused on a biomedical perspective and, even if RLS causes significantly decreased quality of life (Broström et al., 2024), only a few studies have tried to gain an in-depth perspective of how symptoms are featured and expressed (Holzknecht et al., 2020, 2022), as well as how RLS treatment impacts on the life situation (Harrison et al., 2021). This study fills these gaps in the current literature by adding a deeper holistic understanding of how patients experience and handle their symptoms in relation to various human needs is needed, as well as an understanding of how self-care and pharmacological treatment impact these needs. One way to gain such an understanding is to practice Maslow’s hierarchical theory of the five human needs (Maslow, 1943), which are often depicted as hierarchical levels within a pyramid. From the bottom of the hierarchy upwards, the needs are physiological, safety and security, belongingness and love, esteem, and self-actualization (Figure 1, Maslow, 1943).

The model can be used in a structured and systematic way to understand what challenges patients with RLS experience in everyday life. It has been used in many contexts and care situations (e.g., Xu et al., 2021), but it has not been used previously to analyse how patients with RLS sustain their human needs during their everyday life. Understanding facilitators and barriers affecting the fulfilment of each level of human needs, as described by patients with RLS, will increase healthcare professionals’ awareness of RLS as a potential diagnosis, and will help them to design a holistic care approach, when a patient seeks help for problems related to their RLS. It can also help to fine-tune existing screening instruments and develop new patient-reported outcome measures for use in initiating and evaluating pharmacological treatment and self-care interventions for RLS (Fulda et al., 2021). To our knowledge, only one study, using focus groups, has explored the holistic perspective from the everyday life experiences of patients with RLS (Harrison et al., 2021). However, there is lack of studies based on qualitative interviews which has been conducted to determine the facilitators and barriers affecting the fulfilment of each level of human needs, as experienced by patients with RLS. Therefore, the aim of this study was to determine and describe how patients with RLS experience their everyday life, with a focus on facilitators and barriers related to Maslow’s hierarchical theory of the five human needs.

## Methods

### Design

A descriptive deductive design was utilized and methodologically carried out according to qualitative content analysis (Graneheim & Lundman, 2004) and to the COREQ checklist.

### Participants

The study population was derived from a nationwide RLS patient organization with about 1500 members. All members of the organization were invited to participate in a questionnaire-based survey on RLS with the following inclusion criteria: age over 18 years, having been diagnosed and treated for RLS, ability to speak and understand Swedish, and ability to provide written informed consent. Of the 788 members who gave written informed consent to participate in the survey and who returned questionnaires, 472 (60%) expressed their interest in being contacted for a follow-up qualitative in-depth interview. Twenty-eight participants were then strategically selected by the interdisciplinary research team (which included physicians, nurses, and sociologists) to achieve a clinically sound variation based on socio-demographic and situational data: e.g., gender, age, education level, cohabitation, comorbidity and pharmacological treatment (Table II). All 28 who were asked agreed to participate. Ethical approval was obtained from the Swedish Research Council (Dnr: 2022–01515–01).

**Table II.** Socio-demographic and situational data of the patients with RLS (*N* = 28).

Variables	Value
**Gender**, female, n (%)	16 (57)
**Age** (years), mean (range)	67,6 (39–89)
**Educational level**, n (%)	
9 years or below	3 (11)
12–13 years	11 (39)
University	14 (50)
**Civil status**, n (%)	
Married/Living together	20 (71)
Unmarried and living alone	3 (11)
Divorced/widower and living alone	5 (18)
**Smoking**, n (%)	
Yes, n (%)	1 (4)
**Alcohol**, n (%)	
Never uses alcohol	8 (29)
Uses alcohol 2–3 times or more/week	11 (39)
**Comorbidity**, n (%)	
Renal disease	0 (0)
Parkinson’s disease	0 (0)
Multiple sclerosis	0 (0)
Migraine	3 (11)
Iron deficiency	4 (14)
**Pharmacological treatment**, n (%)	
Dopamine agonists	22 (80)
Opioids	8 (29)
α2δ Ligands	7 (25)
Dopa/derivates	5 (20)
Iron supplement	3 (11)

### Data collection

A semi-structured interview guide (Table III) was developed by the interdisciplinary research team, which had extensive competence regarding the treatment of patients with RLS and qualitative content analysis. The interview guide, which was developed based on a deductive holistic approach, was discussed, and scrutinized according to feedback from four patients with RLS of different ages, and minor changes were made (e.g., in the language of the questions and the level of detail of follow-up questions) before the data collection began. The interviews were conducted via telephone during June and November 2022 by three members of the interdisciplinary research team (i.e., two nurses and one sociologist). The procedure began with the participant receiving both written and verbal information about the study and voluntary participation. Thereafter, the participants were given time to ask questions and reflect on their participation. Written and verbal informed consent was obtained from all participants. To assure the confidentiality of the participants, pseudonyms were given the participants to avoid identifying their identity. The interviews, lasting 45–90 minutes, were audio-recorded.

**Table III.** Examples of questions from the semi-structured interview guide.

Examples of questions	Examples of probing questions
Would you please share your experiences of what a typical day might look for you?	Could you please provide further explanation on the occurrence and experiences of symptoms through the day, fatigue and their effect on mood?
Would you please share your experiences of what a typical night might look for you?	Can you provide further explanation regarding the occurrence and experiences of nocturnal symptoms and how they affect your sleep?
Can you describe how you experiences your life situation with RLS?	Can you explain that further relate to your work, family, social life, health, lifestyle and living conditions?
Can you describe how you perceive the support that has been given to you?	Can you provide further explanation about the specifics of the support you have received, including how it was given, when it was given, and the quality of the support you have received?

### Data analysis

**Figure 2.**
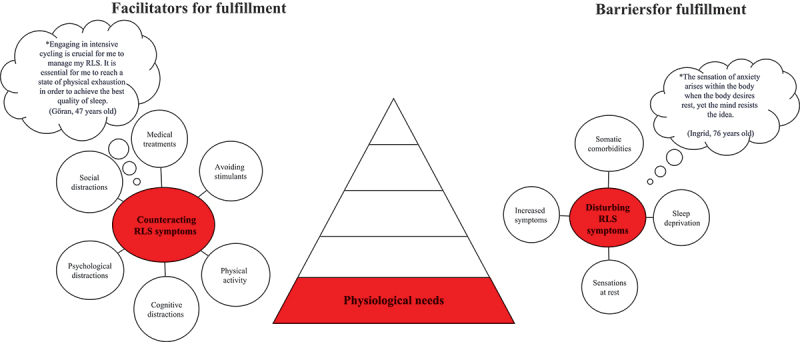
Illustration of facilitators and barriers based on Maslow’s physiological needs as described by people living with rest legs syndrome (*N* = 28).

**Figure 3.**
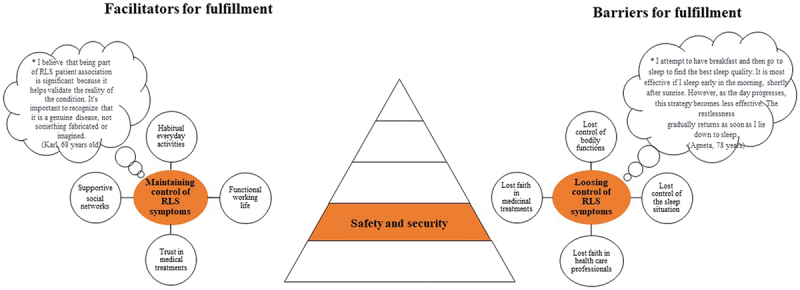
Illustration of facilitators and barriers based on Maslow’s safety and security needs as described by people living with rest legs syndrome (*N* = 28).

**Figure 4.**
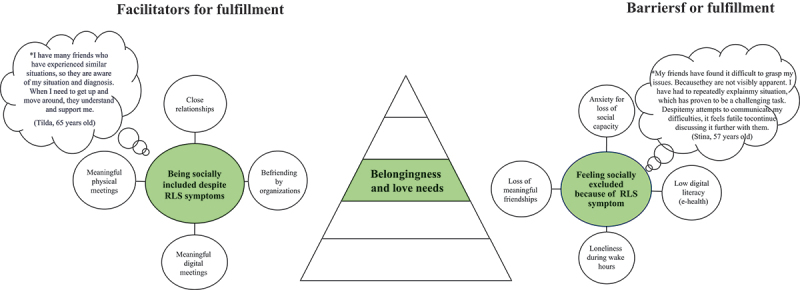
Illustration of facilitators and barriers based on Maslow’s belongingness and love needs as described by people living with rest legs syndrome (*N* = 28).

**Figure 5.**
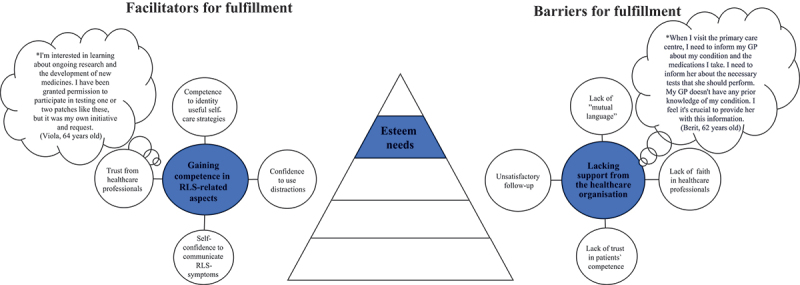
Illustration of facilitators and barriers based on Maslow’s esteem needs as described by people living with rest legs syndrome (*N* = 28).

**Figure 6.**
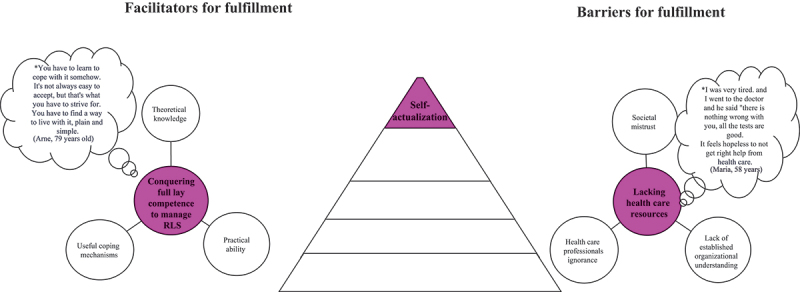
Illustration of facilitators and barriers based on Maslow’s self-actualization needs as described by people living with rest legs syndrome (*N* = 28).

Verbatim transcripts of all interviews were produced, resulting in 314 A4 pages of single-spaced text in 12-point Times font. The deductive analysis was performed by the interdisciplinary research team based on Maslow’s hierarchical theory of five human needs (Figure 1, Maslow, 1943). Firstly, the transcribed interviews were carefully read and checked for accuracy by the interviewer. Secondly, all the interviews were read several times by all interdisciplinary research team members with the intention to get a sense of the whole and to identify statements (i.e., meaning units). Thirdly, these meaning units were read repeatedly by the interdisciplinary research team, then compared and clustered into relevant human needs related to Maslow´s hierarchical theory of five human needs. Fourthly, meaning units were developed at each level and then divided into facilitators and barriers. Finally, all members of the interdisciplinary research team engaged in discussions to establish a category system. The discussions continued until consensus was reached. The system included categories and subcategories (illustrated in Figures 2–6 and the text in the results section) describing facilitators and barriers for fulfilment according to human needs.

*Bubble presents quotes from patients.

*Bubble presents quotes from patients.

*Bubble presents quotes from patients.

*Bubble presents quotes from patients.

*Bubble presents quotes from patients.

## Results

Facilitators and barriers affecting the fulfilment of Maslow’s hierarchical theory of the five human needs are described in Figures 2–6.

### Physiological needs

#### Counteracting RLS symptoms

Counteracting RLS symptoms was described as a facilitator for the fulfilment of physiological needs in everyday life, and this category comprised six subcategories (Figure 2). Regular use of RLS-specific medications, avoidance of stimulants, being in a quiet environment without being disturbed to catch up on a few hours of sleep, and constant movement, such as cycling, and exercise, were described as essential to counteract RLS symptoms during daytime. One or two hours of rest or sleep after breakfast and taking medication for RLS were used to get an acceptable amount of sleep. Cognitive (e.g., listening to music), psychological (e.g., being active in social activities), and social (e.g., meeting other people) distractions were used to take focus away from RLS symptoms.

#### Disturbing RLS symptoms

Disturbing RLS symptoms was described as a barrier to fulfil physiological needs, and this category included four subcategories: somatic comorbidities, sleep deprivation, sensations at rest and increased symptoms (Figure 2). These symptoms led to frustration and anxiety during the afternoon and at night and when going to bed, making it difficult to sleep. A crawling sensation, pain in the legs, excessive fatigue before sleep, and irritability caused loneliness during the night since those living with a partner left the bed to avoid disturbing the partner. During the daytime, symptoms (e.g., fatigue, anxiety) often started with sensations while lying in bed or resting.

### Safety and security needs

#### Maintaining control of RLS symptoms

Maintaining control of RLS symptoms was described as a facilitator for fulfilling safety and security needs, and this category comprised four subcategories (Figure 3). Habitual everyday activities, functional working life, trust in medical treatments and supportive social networks were used. Medication was essential and had to be taken regularly to avoid RLS symptoms. The participants followed a strict timetable for their treatment (e.g., intake of medication) to maintain control and create a functional everyday life. A functioning work situation, even if suffering from RLS symptoms, was based on understanding and trust from the employer, which enabled adaptation to the individual’s needs, e.g., through digital meetings from home. A sense of being connected to and contributing to something bigger was revealed, which enriched social contacts, created safety, and momentarily diverted thoughts from the RLS symptoms.

#### Losing control of RLS symptoms

Losing control of RLS symptoms was described as a barrier to fulfilling safety and security needs and this category comprised four subcategories (Figure 3). Lost control of bodily functions and the sleep situation, as well as lost faith in healthcare professionals and medical treatment were described. A sensation like “a world war inside the body and head” based on the perceived loss of control of the arms and legs led to poor sleep quality and experiences of feeling wired and tired during the daytime. Faith in healthcare was lost, especially in primary healthcare since its responsibility for the patient’s sleep-related problems was described as missing. This led to self-adjustment of the medical regime due to limited availability for follow-up with the physician. This was especially difficult for those who lacked knowledge about RLS and led to a lack of trust in medical treatments.

### Belongingness and love needs

#### Being socially included despite RLS symptoms

Being socially included despite RLS symptoms in everyday life was described as a facilitator for fulfilling belongingness and love needs and this category comprised four subcategories (Figure 4), namely close relationships, befriending by organizations, meaningful digital meetings, and meaningful physical meetings. Close and long-term relationships with openness about RLS led to disease- and symptom-related support, understanding and adaptation from family and friends, simplified new relationships, new meaningful digital and physical meetings, and relationships beyond existing social networks to other organizations. The RLS patient organization opened a social world where others with the same condition could make new friends. Support members in the patient organization took on the role of friendship facilitator, which evolved into supportive and close affiliations that were maintained by phone or email.

#### Feeling socially excluded because of RLS symptoms

Feeling socially excluded because of RLS symptoms was described as a barrier to the fulfilment of belongingness and love needs and this category included four subcategories: anxiety about the loss of social capacity, low digital literacy, loneliness during waking hours and loss of meaningful friendships (Figure 4). A constant sensation of anxiety and concern about the loss of social capacity because of RLS symptoms led subjects to avoid relationships, which was based on experiences of being invisible and misunderstood both in society and the digital world, because RLS was unnoticed by others, even healthcare professionals. Digital networks were hard to access because of the low degree of digital literacy and the lack of skills needed to use different social media. This led to social exclusion, loneliness during waking hours, loss of meaningful friendships, frustration, and a negative view of everyday life.

### Esteem needs

#### Gaining competence in RLS-related aspects

Gaining competence in RLS-related aspects was described as a facilitator for fulfilling esteem needs in everyday life and this category included four subcategories (Figure 5), namely competence in identifying useful self-care strategies, confidence in using distractions, self-confidence in communicating RLS symptoms, and trust in healthcare professionals. Patients with RLS gained competence and self-confidence in identifying, exploring, and utilizing various self-care strategies and distractions, such as bicycling, gardening, to manage RLS symptoms during the day and at night. They communicated these strategies to healthcare professionals based on their own self-knowledge and experiences, thereby taking control of their RLS symptoms, and boosting their self-esteem. They used their own initiative by writing referrals to specialist clinics, contacting private clinics to submit blood samples (e.g., a test of ferritin), or adjusting their medication regimen based on self-esteem and competence.

#### Lacking support from the healthcare organisation

A lack of support from the healthcare organization was described as a barrier to fulfilling esteem needs and this category included four subcategories: lack of mutual language, lack of faith in healthcare professionals, lack of trust in the medical competence of healthcare professionals, and unsatisfactory follow-up (Figure 5). A language barrier and lack of trust in healthcare professionals were described based on the perception that healthcare professionals were unresponsive and did not provide feedback about the medical treatment, but there was also a lack of coordination in planning the treatment, insufficient knowledge, and information about RLS symptoms, and failure to consult with relevant organizations for advice. This was expressed as feeling a lack of trust from healthcare professionals in one’s own competence (i.e., the patients’ competence), and unsatisfactory follow-up regarding the treatment, RLS symptoms, and the overall picture of the condition.

### Self-actualisation needs

#### Conquering full lay competence to manage RLS

Achieving full lay competence to manage RLS was described as facilitator for fulfilling self-actualization needs in everyday life and comprised three subcategories: theoretical knowledge, practical ability, and useful coping mechanisms (Figure 6). An in-depth understanding of the disease and its treatment was described as essential to be able to use all lay- and care-related treatment resources to solve problems related to patients own treatment in an optimal way, empowering and motivating patients to continuously seek out new or helpful information and ideas on potential coping mechanisms.

#### Lacking healthcare resources

A barrier to fulfilling self-actualization needs was described as a lack of healthcare resources and this category comprised three subcategories: societal mistrust, lack of established organizational understanding and ignorance among healthcare professionals (Figure 6). The focus was on handling RLS symptoms; time and concern for others in the social network disappeared when the RLS symptoms took over, leading to societal mistrust. The lack of established organizational understanding and ignorance among healthcare professionals about the obstacles in everyday life with RLS contributed to a reduced quality of life.

## Discussion

Our study determined and described a variety of facilitators and barriers to the fulfilment of human needs (Maslow, 1943). Apart from Harrison et al. (2021), who described experiences of living with RLS, this is the first qualitative methodologically analysed study to adopt a holistic perspective when determining these aspects. RLS care is complex, and as Fulda et al. (2021) have pointed out, an improvement is required to fine-tune ways to identify patients and evaluate their needs (e.g., by validated instruments), and to develop suitable holistic interventions. The experiences provided by patients with RLS from the current study can be used to provide a holistic perspective.

We found that fulfilment of physiological needs was achieved through adapting medication, engaging in regular physical activity, creating a calm environment, and having access to cognitive, psychological, and social distractions. On the other hand, factors such as somatic comorbidities, sleep deprivation, sensations at rest, and an increase in symptoms, which led to a loss of control of RLS symptoms, were described as barriers. Our findings are consistent with previous research conducted from the perspective of patients, which indicates that healthcare professionals have a limited understanding of RLS symptoms and do not prioritize the management of RLS symptoms (Harrison et al., 2021). In a clinical situation, when the time to provide in-depth information is limited, it might be difficult for the physician and patient to agree upon the best treatment option, partly due to varying anamnestic descriptions by patients (Holzknecht et al., 2022) or due to time constraints. In the present study, patients with RLS said that the lack of knowledge and understanding about RLS posed a significant barrier to fulfilling their esteem needs. Additionally, patients described a lack of confidence in healthcare professionals, as physicians often demonstrated a disinterest in gathering information from them. This disinterest undermined the foundation for person-centred care (Dewing & McCormack, 2017), where patients should be actively involved in their own care and in decisions regarding their treatment (Swanson, 1991). Furthermore, we found that this disinterest displayed by healthcare professionals not only caused frustration among patients, but it also prompted them to independently seek knowledge and competence in RLS-related matters through online resources. A growing movement in healthcare referred to as “humanising of healthcare”, which started in the late 20th century, focuses on the person at the centre of healthcare decisions (McCormack et al., 2017). Such a perspective requires different approaches in research to knowledge development. In comparison with standardized evidence-based medicine, underpinned by positivism, a person-centred approach requires a more eclectic knowledge base. By adopting person-centred care, attentiveness and dialogue, empowerment and participation, and critical reflexivity (Jacobs et al., 2017) are necessary for patients with RLS to be involved in their care as creative and resourceful actors. In this study, we found that all human needs (Maslow, 1943) manifest in various ways through patients actively combating the everyday problems caused by RLS. Therefore, holistic, and person-centred interventions are needed on all levels (i.e., personal, organizational, and social/political) to capture all aspects of everyday life. Furthermore, we found that this disinterest displayed by healthcare professionals not only caused frustration among patients, but it also prompted them to independently seek knowledge and competence in RLS-related matters through online resources. Health literacy is “the ability to seek, find, understand, and appraise health information from electronic sources and apply the knowledge gained to addressing or solving a particular health concern” (Ratzan & Parker, 2000). Our findings regarding use of online resources are consistent with previous research, conducted from the perspective of patients with chronic obstructive pulmonary disease (COPD), where knowledge about one’s own disease was indicative of higher quality of life (Stellefson et al., 2019; Ture et al., 2020). Therefore, understanding the individual’s ability to obtain relevant information becomes important. A plausible instrument to evaluate competence in health information seeking behaviour in RLS is the eHealth Literacy Scale (Norman & Skinner, 2006). We also found that esteem needs were impeded by the lack of support from healthcare. Factors such as lack of mutual language, lack of faith in healthcare professionals, healthcare professionals’ perceived lack of trust in patients’ competence, and unsatisfactory follow-up prevented patients with RLS from feeling confident in managing their symptoms and communicating their needs effectively. Finally, the lack of resources was identified as a barrier to fulfilling the fifth human need, self-actualization (Maslow, 1943). This was attributed to societal mistrust, lack of established organizational understanding, and ignorance among healthcare professionals.

Shared decision-making (SDM), which is a two-way process to help the physician and patient to agree on interventions (Elwyn et al., 2016) might be an applicable measure to handle some of these above-described care-related barriers around pharmacological treatment (Lv et al., 2021). However, clinical appointments are in most cases limited by time, which makes communication between patients and healthcare professionals extra important (National Quality Forum, 2017) to make SDM work. Therefore, the use of the four-habits communication model, which aims to quickly build trust between patient and physician, achieves an effective exchange of information (Frankel & Stein, 2001). The physician should “invest in the beginning”, “find out the patient’s perspective”, “show empathy” and “invest in the end” to agree on treatment. When using SDM in an appointment, the physician needs to carefully listen to the patient and consider “*What is he saying*?” and not decide for himself “*His needs are xx*” (Oerlemans et al., 2021). To avoid decisional conflicts, the patient’s own role in making decisions needs to be considered (Légaré et al., 2008), with emphasis on how different patterns of the disease impact everyday life at different ages (Didato et al., 2020). Short instruments are preferred in clinical situations to measure SDM (e.g., CollaboRATE, three items, Elwyn et al., 2016) or decisional conflict (e.g., SURE, four items, Légaré & Thompson-Leduc, 2014). Both these instruments have recently been validated in RLS-patients with good results (Björk et al., 2023) and could be used in clinical situations to evaluate SDM.

Seeking information on the Internet, described as a good ability to acquire and utilize information through digital platforms, was mentioned as a facilitator to maintaining esteem needs (Maslow, 1943). This finding is consistent with previous studies in various healthcare domains, where e-health has become an integral part (Gordon & Hornbrook, 2016; Hordern et al., 2011). It also highlights the importance of fostering e-health literacy (Paige et al., 2017). Therefore, it is crucial for physicians and other healthcare professionals to actively incorporate e-health literacy into their practice, considering its significance in the future development of RLS care and treatment. Internet-based cognitive behavioural therapy (ICBT), i.e., therapy provided through a computer, or a mobile device, is nowadays a common solution to improve accessibility (Kumar et al., 2017), and has e.g., in insomnia, proven to be as good as cognitive behavioural therapy (CBT) delivered face-to-face by a therapist (Esfandiari et al., 2021). CBT has recently been tested in a small-scale RLS study with good results on sleep problems (Song et al., 2020). ICBT has not been used in an RLS context, but could if adapted to the situation, be a potential solution to increase accessibility, as well as the delivery of relevant information and suitable holistic behavioural change interventions. Furthermore, ICBT has been used with promising results in various other conditions that are characterized by chronic symptoms (e.g., pain or tinnitus). The establishment of changed habits might be another way to cope with burdensome RLS symptoms. Gardner (2015) states that habits (i.e., routines that are practised regularly) are automatic, un-reflected reactions to an internal or external trigger, acquired through repetition of a behaviour in the presence of these triggers. In an RLS context, cues that trigger action (e.g., for taking medication) can be anything from a sensation (e.g., perceptions of RLS symptoms), a time (e.g., the evening), or a physical location (e.g., the bedroom). In the present study, habitual everyday life activities were mentioned as facilitators for the fulfilment of safety and security needs (Maslow, 1943). If the physician can identify triggers/stressors, then support can be implemented to try to break a negative habit (e.g., stressful thoughts when symptoms start in the evening) and create new positive and helpful habits (e.g., use of distraction, adapting medication intake, or increasing use of self-care actions). As the habit has been formed, maintenance of the behaviour becomes less reliant on conscious attention and memory processes and instead becomes automatic (Gardner, 2015). By identifying and addressing facilitators for the development of positive habits and how to cope with RLS symptoms, healthcare professionals might promote the fulfilment of human needs and improve the quality of life for patients with RLS.

By determining and describing the everyday lives of patients with RLS in relation to Maslow’s hierarchical theory of five human needs (Maslow, 1943), a holistic perspective was revealed. This approach allowed us to capture the experiences of patients with RLS, focusing on their human needs. This study has captured the everyday life of patients with RLS; our findings therefore add new insights and implications to clinical practice. A specific and more nuanced understanding of the everyday life of patients with RLS is important as healthcare professionals sometimes have difficulties looking beyond the diagnosis to consider some of the significant differences in patients’ living situations and capacity to cope. Such awareness, of course, applies not least to a holistic perspective (Fridlund & Baigi, 2014; Sarvimäki & Stenbock-Hult, 1993) where healthcare professionals should be aware of what life is like for patients with RLS and the crucial role that they can play to meet patient’s needs. Moreover, the communities in which patients reside also play an important role. However, this holistic approach requires looking beyond the biomedical model that currently frames the care of patients with RLS. Therefore, future research needs to map the everyday life of patients with RLS, including facilitators and barriers during both day and night. This requires the incorporation of more specific theories, concepts, and designs, both qualitative and quantitative, because there is a lack of in-depth knowledge in this area. Existing research has mostly focused on medications, which highlights the need for studies that explore various everyday life aspects e.g., quality of life. Therefore, it is crucial to conduct qualitative studies that explore and describe self-care measures to gain a comprehensive understanding of the experiences and needs of patients with RLS through holistic and person-centred interventions on all levels (i.e., personal, organizational, and social/political). In addition, a validated instrument to measure frequency and benefit of self-care actions on RLS symptoms is also missing and needs to be developed and psychometrically tested. Furthermore, there is a need to validate instruments that are more oriented towards all aspects in everyday life (e.g., Fridlund et al., 2015), and communication (e.g., the four-habits communication model, Frankel & Stein, 2001).

### Study limitations and strengths

There are several limitations to be considered. Firstly, the participants were selected from a patient organization. This could impact the findings, as they may have been more engaged in their RLS diagnosis and care compared to persons not involved in a patient organization. However, a particular strength is that our data is based on 28 participants, a relevant and plausible number of participants with respect to a strategic selection in a qualitative study, with variations in age, gender, and education, that describe and illuminate patients with RLS. Patients with a varied symptom burden and treatment regimens are included. This increases the credibility and transferability of the results. Secondly, the use of telephone interviews is often seen as a less attractive alternative to face-to-face interviewing due to the absence of visual cues, resulting in the loss of contextual and nonverbal data that could compromise the probing and interpretation of responses (Novick, 2008). However, telephone interviews allowed the participants to feel relaxed and able to disclose sensitive information about their RLS diagnosis. Thirdly, a limitation could be the potential influence of the three interdisciplinary research team members who conducted the interviews. However, the risk of discrepancies in our data collection was mitigated by extensive discussions in the interdisciplinary team, which allowed for different perspectives on the issue under study and increased dependability and confirmability. Fourthly, the deductive approach was based on Maslow’s hierarchical theory of five human needs (Maslow, 1943). Even if the deductive approach provides a holistic approach, it can limit the understanding of everyday life. Finally, it is important to acknowledge that while Maslow’s hierarchy of needs (Maslow, 1943) has been widely used in the behavioural sciences, there are different perspectives and criticisms regarding its application. One criticism is the difficulty in distinguishing between the different needs and the notion that individuals must satisfy lower-level needs before progressing to higher-level needs (Kenrick et al., 2010; McLeod, 2018). Our analysis revealed that identifying descriptions of facilitators and barriers for the first three levels of needs was relatively easy, while the higher levels became progressively more challenging. This methodological limitation arises from the complexity of connecting these levels to the underlying theory and achieving the higher levels of needs. Nonetheless, one strength of our analysis was the rich variation and comprehensive descriptions at each level, which added depth, and increased credibility and transferability to our findings.

## Conclusions

This study highlights the importance of addressing the facilitators and barriers faced by patients with RLS in fulfilling their basic needs. There is a need for holistic and person-centred interventions on all levels (i.e., personal, organizational, and social/political) that address the physiological, psychological, and social needs of patients with RLS, as well as the need for education and training of healthcare professionals to enhance their understanding of RLS and the provision of effective care. Holistic and person-centred interventions, including facilitators for the fulfilment of physiological, psychological, and social needs could help healthcare professionals to provide holistic care.
